# *in vitro* comparison of surgical guide technologies on osteotomy precision in anterior maxillary implant sites

**DOI:** 10.6026/973206300222514

**Published:** 2026-04-30

**Authors:** Khwairakpam Chaoton Singh, Bhumika Sehdev, Ravindrakumar Prakash Gedam, Manoj Manohar, Sameer Gupta, Kunal Banka

**Affiliations:** 1 Department of Oral and Maxillofacial Surgery, Yogita Dental College & Hospital, Ratnagiri, Maharashtra, India; 2 Department of Periodontology & Implantology, DJ College of Dental Sciences and Research, Gaziabad, Uttar Pradesh, India; 3 Department of Oral Pathology and Microbiology, Dr.Rajesh kambe Dental College and Hospital, Akola, kanheri, Sarap, Maharashtra, India; 4 Department of Oral and Maxillofacial Surgery, Second Health Cluster, Ministry of Health, Riyadh, Saudi Arabia; 5 Department of Oral & Maxillofacial Surgery, Inderprastha Dental College and Hospital, Ghaziabad, Uttar Pradesh, India; 6 Department of Implantology, Dental clinic Implantologist, Jharkhand, India

**Keywords:** Surgical guide, implant accuracy, osteotomy precision, 3D printing, anterior maxilla

## Abstract

Accurate implant osteotomy positioning in the anterior maxilla remains a clinical challenge because the precision of different surgical guide fabrication methods has not been fully established. Therefore, it is of interest to compare the accuracy of osteotomy preparation using conventional vacuum-formed, desktop 3D-printed and industrially milled surgical guides in standardized anterior maxillary resin models. Thirty identical maxillary resin models with simulated central incisor edentulous sites were allocated into three groups (n=10 each) and osteotomy deviations from a single virtual implant plan were assessed using post-operative CBCT superimposition software for coronal, apical, angular and depth discrepancies. Industrially milled guides showed the lowest mean deviation at both the coronal (0.38 ± 0.14 mm) and apical (0.52 ± 0.18 mm) levels, followed by 3D-printed guides (0.54 ± 0.19 mm and 0.78 ± 0.22 mm), whereas conventional guides showed the greatest deviation. Digitally fabricated guides, particularly industrially milled guides, provided significantly greater osteotomy accuracy than conventional vacuum-formed guides in anterior maxillary implant placement.

## Background:

The clinical context of placing implants in the anterior maxillary territory is one of the most challenging clinical settings in the current implant dentistry both due to the strict esthetic demands and because of the small size of the bone, anatomical limitations and low margin of error in the quest to create a harmonized emergence profiles and ideal prosthetic success [1]. The tiniest displacement of the desired implant location in this area may lead to the damage of esthetics, poor soft tissues, phonological disruptions, biomechanical issues and the necessity of massive corrective prosthetic actions [2]. Therefore, accurate positioning of implants using three-dimensional treatment planning driven by the prosthetics has become a critical requirement to the attainment of predictable outcomes in the esthetic zone [3]. Guided implant surgery was created to fill the gap between virtual implant planning and clinical implantation by transfer of digitally planned implant position to the surgical site using a physical template [4]. Surgical guides are mechanical devices that limit the position, angulation and depth of the osteotomy tool, which improves the accuracy and predictability of implant placement in comparison with freehand methods [5]. The development of surgical guide technology has developed over numerous generations, starting with crudely radiographic templates and vacuum-formed guides, up to the current sophisticated computer- aided design systems and computer-aided manufacturing systems that combine high-quality imaging, computer-based planning and precision fabrication [6]. Historically, simple directional guides have been used in implant surgery with the use of the conventional surgical guides, traditionally manufactured by vacuum-forming materials over stone casts. They are typically created by modifying a piece of thermoplastic sheet over a diagnostic cast upon which the intended implant location is drawn and a guide tube or channel is added to the location of the intended osteotomy [7]. Although this method is comparatively cheap and simple, it is also inherently restricted by the accruing errors involved in the process of taking impressions, cast fabrication, manual positioning of guide tubes and the imprecision of the vacuum-forming process itself [8].

Computer-guided implant surgery has brought the idea of digital workflows, which involve the implementation of cone-beam computed tomography images, advanced planning software and computer-aided manufacturing to create surgical guides with significantly greater precision [9]. In this digital paradigm, two major fabrication technologies have been created, additive manufacturing with 3D printing and subtractive manufacturing with industrial milling. The desktop stereolithography 3D printers have become more affordable and accessible, allowing fabrication of surgical guides at the chairside with biocompatible photopolymer resins [10]. Instead, commercially-milled guides are made by machine milling of polymethylmethacrylate or other biocompatible blanks by means of computer-numerical-controlled milling machines, which is often contracted to dedicate manufacturing facilities [11]. Within every fabrication technology, there are some sources of error associated with it, which can impact the ultimate accuracy of the surgical guide. In 3D printing, dimensional fidelity of the guide may vary depending on the layer thickness, the direction in which the build is made, the positioning of support structures, the shrinkage of the photopolymerization process, post processing procedures and calibration of the printer [12]. Machine precision, tool diameter, material properties and quality of digital design file mostly control the accuracy during milling [13]. The relative contribution of this source of technology to the total osteotomy precision is important as a basis of evidence-based clinical decision-making. Available literature on surgical guide accuracy is massive but is disparate. A number of systematic reviews and meta- analyses have reported on the general accuracy of computer-guided implant surgery with a mean deviation of about 1.0 to 1.4 mm at the implant platform and 1.2 to 1.7 mm at the apex and angular deviation of 3°C to 5°C [14]. Nevertheless, a majority of these studies have considered guided surgery as one category and few studies have compared actual precision of various guide manufacturing methods in controlled experimental conditions [15]. Moreover, most of the comparative studies that have been conducted have been done on posterior implant sites where the effects of slight changes in position are not as critical clinically in the anterior esthetic area [16]. The anterior maxilla has special problems such as thin labial bone plates, closeness to the nasopalatine canal, high smile line visibility and the extreme significance of the position of facial-palatal implants in providing natural soft tissue emergence profiles [17]. Specific anatomically and prosthetically challenging investigations of guide accuracy are missing in this area. The other major literature gap is related to the comparison of digital fabricated guides to traditional vacuum-shaped guides. Although the positive effect of computer- guided surgery over freehand surgery has been very well-established, the incremental accuracy advantage of computer-generated guides versus hand-crafted traditional guides has not been clearly quantified [18]. Therefore, it is of interest to compare the osteotomy accuracy of conventional vacuum-formed, desktop 3D-printed and industrially milled surgical guides in anterior maxillary implant placement.

## Materials and Methods:

## Study design:

This comparative experimental research was an *in vitro* comparative study that was carried out at the digital dentistry and implant research lab in the Faculty of Dentistry. The precision of the osteotomy was assessed in comparison of planned and achieved positions of implants using three surgical guide fabrication techniques.

## Master model fabrication:

The master template would be a commercially available fully dentate maxillary resin typodont model (Nissin Dental Products, Kyoto, Japan). In order to model an anterior edentulous site, the right central incisor tooth was extracted and the alveolar socket replaced with autopolymerizing acrylic resin to look like a healed extractions site with type II bone density. The left over ridge size was scaled in such a way that it displayed a sufficient volume of bone in which the implants could be placed (buccopalatal width: 7.5 mm, mesiodistal width: 8.0 mm, available height: 14.0 mm). A high-precision silicone mold (Elite Double 32, Zhermack, Italy) and polyurethane resin (Exakto-Form, Bredent, Germany) that replicated the density of D2 bone with a cortical layer of thickness 2 mm and cancellous core density 0.32 g/cm3 were used to produce 30 identical copy models of the modified master model. All replicas were checked under the eye of digital calipers to ensure that the replica had the same dimensions as the master model and those that were found to be more than 0.2 mm away were discounted and remade.

## Virtual planning and CBCT scanning:

A cone-beam computed tomography unit (Planmeca ProMax 3D Mid, Planmeca Oy, Helsinki, Finland) with standardized parameters (90 kV, 10 mA, voxel size 0.15 mm, field of view 8 x 8 cm) was used in scanning the master model. Furthermore, a laboratory optical scanner (3Shape E4, 3Shape A/S, Copenhagen, Denmark) was used to give a high-resolution surface scan with an accuracy of 4 5m. The CBCT data (DICOM format) and surface scan data (STL format) were loaded into implant planning software (coDiagnostiX, Dental Wings, Montreal, Canada) and overlaid with automated registration algorithms with manual fine-tuning. One virtual implant (4.0 mm diameter x 12.0 mm length) was to be placed in the right central incisor area in accordance with the following established principles of the prosthetically driven planning: the implant apical level 3 mm above the proposed gingival margin, mesiodistally located within the edentulous span, with the platform of the implant 2 mm palatally to the proposed buccal emergence point. The reference standard of the accuracy comparison of all further comparisons was the three-dimensional coordinates and the angular orientation of the designed implant that were recorded and locked down.

## Design and fabrication of surgical guide:

One type of surgical guide was designed in the planning program according to the virtual implant plan. The guide was made by following a tooth-supported template, bilateral occlusal rests and one metallic guide sleeve (5.0 mm outer diameter, 2.2 mm internal diameter according to the final drill, 5.0 mm sleeve height) at the scheduled osteotomy location. Both digital fabrication approaches were done using the same file (STL format) of the digital guide design to remove variability due to design.

## Group 1: Vacuum-Formed Guide of the conventional type (n = 10):

A type IV dental stone cast was poured by taking an impression of the master model with polyvinyl siloxane material (Elite HD+, Zhermack, Italy). A vacuum-forming machine (Erkoform 3d, Erkodent, Germany) was used to cover the cast with a thermoplastic sheet (Erkodur, Erkodent, Germany, 2-mm). The target location of the implant was transferred to the guide with the help of laboratory surveyor and radiographic markers based on the master model CBCT scan. The planned position was fixed with the help of the autopolymerizing acrylic resin of the stainless steel guide tube (5.0 mm outside diameter, 2.2 mm inside diameter). On the master model, guide fit was checked.

## Group 2: Desktop 3D-Printed Guide (n = 10):

The digital guide was exported as a stereolithography3D model in a form of STL and printed with a desktop stereolithography 3D printer (Form 3B+, Formlabs Inc., Somerville, MA, USA) on biocompatible surgical guide resin (Surgical Guide Resin V1, Formlabs). Print settings were a layer thickness of 50 u and a build orientation that allowed the most accurate sleeve (guide was at a 45 degree angle with respect to the build platform). Guides were then printed followed by post-processing (washing) in an automated wash (Form Wash, Formlabs) with 10 minutes in 99% isopropyl alcohol, after which they were post-cured in a UV curing unit (Form Cure, Formlabs) to 60°C in 20 minutes. Guide sleeves Group 1 sleeves were pressed into the printed guide.

## Group 3: Industrially Milled Guide (n=10):

The same STL file was given to a professional industrial fabrication facility to be fabricated in a five axis CNC milling machine (Datron D5, Datron AG, Germany). Polymethylmethacrylate blanks (PMMA) were milled to be used as guides. Industrial precision was applied in the insertion of metal guide sleeves in controlled press-fit conditions. Completed guides were supplied with verification certificates as regards to the dimensional accuracy within the tolerance mentioned by the manufacturer of ± 0.05 mm.

## Osteotomy and implant placement:

All the osteotomies were done by one trained operator that was not aware of any particular hypotheses of the research. Each resin model was placed on the surgical guide and its fit was checked both visually and tactilely. Guidance was made by the manufacturer with the guided surgery instrument kit (pilot drill, 2.0 mm twist drill, 2.8 mm twist drill, 3.5 mm twist drill) sequentially in the guide sleeve at 1200 rpm under vigorous saline irrigation. A guided implant driver was then used to insert an implant analog (4.0 mm x 12.0 mm, titanium alloy) to full depth into the guide. Following the placement of the implants, the surgical guide was taken away and the position of the implant analog was checked to ascertain full seating. A coded identifier was put on each model to keep the operators blinded in their subsequent analysis.

## CBCT and accuracy analysis postoperative:

Scans of each of the analog of the implant placed were taken on the same CBCT unit with the same parameters as the preoperative scan. CBCT data of the postoperative stage were imported in the planning software and overlaid on the preoperative virtual plan in voxel-based rigid registration.

The parameters of deviation measured by one calibrated examiner who was blinded to group assignment were the following:

[1] Coronal (platform) deviation (mm): Three dimensional linear distances between the designed and obtained centers of the implant platforms.

[2] Apical deviation (mm): Linear distance of the planned and actual position of the apex of the implant in three dimensions.

[3] Angular deviation (): Angle between planned and actually achieved long-axis of the implant.

[4] Depth deviation (mm): The difference between the planned and measured implant depth along the implant axis (negative = shallower than planned; positive = deeper than planned)

[5] Buccopalatal deviation (mm): Horizontal deviation in the buccopalatal direction at the platform level.

[6] Mesiodistal deviation (mm): Horizontal deviation at the platform at the mesiodistal.

Each of the measurements was carried out twice after a period of two weeks and the combination of the two measurements was analyzed. The intraclass correlation coefficient was determined to determine the intra-examiner reliability.

## Statistical analysis:

The analysis of data was performed with the help of SPSS version 27.0 (IBM Corp., Armonk, NY, USA). Mean, standard deviation, median, minimum, maximum = descriptive statistics were computed. The Shapiro-Wilk test was used to test normality. Between-group comparisons of continuously distributed variables that were normally distributed were done using one-way ANOVA.

## Results:

All thirty surgical guides seated satisfactorily on their respective resin models. No guide fractures, sleeve dislodgement, or osteotomy- related model damage occurred during the procedures. The intraclass correlation coefficient for intra-examiner reliability was 0.96, indicating excellent reproducibility. Three-dimensional linear deviations are presented in Table 1 (see PDF). One-way ANOVA showed statistically significant differences among the three groups for coronal, apical, buccopalatal and mesiodistal deviations (p < 0.001). The conventional guides showed the greatest deviations at all measured levels, followed by 3D-printed guides, while milled guides demonstrated the least deviation. Coronal deviation was highest in the conventional group (1.24 ± 0.31 mm), followed by the 3D-printed group (0.54 ± 0.19 mm) and milled group (0.38 ± 0.14 mm). A similar pattern was observed for apical deviation, with values of 1.67 ± 0.42 mm, 0.78 ± 0.22 mm and 0.52 ± 0.18 mm, respectively. Angular and depth deviations are presented in Table 2 (see PDF). Angular deviation was significantly higher in the conventional group (4.87 ± 1.34°C) compared with the 3D-printed group (2.14 ± 0.78°C) and milled group (1.53 ± 0.62°C) (p < 0.001). Depth deviation followed the same trend, with the highest deviation in conventional guides (0.92 ± 0.34 mm), followed by 3D-printed guides (0.43 ± 0.18 mm) and milled guides (0.31 ± 0.14 mm) (p < 0.001). Post-hoc pairwise comparisons are summarized in Table 3 (see PDF). The conventional group showed significantly greater coronal, apical, angular, depth, buccopalatal and mesiodistal deviations compared with both the 3D-printed and milled groups (p < 0.05). Between the 3D-printed and milled groups, statistically significant differences were observed only for apical deviation (mean difference: 0.26 mm, p = 0.014) and angular deviation (mean difference: 0.61°C, p = 0.042). Differences in coronal deviation, depth deviation, buccopalatal deviation and mesiodistal deviation were not statistically significant. Overall, milled surgical guides demonstrated the highest accuracy, followed by 3D-printed guides, whereas conventional guides showed the lowest accuracy. The superiority of milled guides over 3D-printed guides was statistically significant only for apical and angular deviations.

## Discussion:

The current *in vitro* experiment compared the accuracy of the osteotomy of three types of technologies of the fabrication of surgical guides in simulated anterior maxillary implantation sites. These findings provided strong evidence on accuracy between the three types of guides, as the guides milled industrially had the most accuracy, the 3D-printed guides on desktops had the next highest accuracy and the traditional vacuum-formed guides exhibited significantly high deviations in all measured factors. The null hypothesis is rejected by these findings and it gives evidence-based advice on the choice of suitable guide fabrication techniques especially of the challenging esthetic zone. This is because the error introduced by the fabrication workflow through the analog process has accumulated in the conventional vacuum-formed guides yielding significantly less accurate results than either of the two types of digital guides. The dimensional error is introduced at each stage of the traditional process impression taking, stone cast fabrication, thermoplastic sheet adaptation and manual guide tube positioning, which are cumulative to create the ultimate positional error [19]. The vacuum-forming procedure per se creates distortion, being the stretching and contraction of materials when cooled to allow adaptation to the dental arch that are less than optimal and compromised stability in the process of preparing osteotomy holes [20]. Moreover, the manual insertion of guide tube depends on the operator judgment and laboratory measurements, not as precise as computed sleeve positioning that can be obtained by digitally computing [21]. The average coronal deviation of 1.24 mm and apical deviation of 1.67 mm with traditional guides in this study are similar with the values found in other studies, which compare non-digital surgical guides. Past research has documented coronal misalignment of 1.0 to 2.0 mm and apical misalignment of 1.2 to 2.5 mm using traditional methods of guides [22]. Although these deviations might be clinically reasonable in more posterior areas when the bone volume is sufficient and esthetic demands are lower, they are of great concern in the anterior maxilla, where a standard deviation as little as 1 mm in the buccopalatal direction can lead to the rupture of the buccal plates, exposing the threads of the implant, or an unsatisfactory emergence profile [23]. The quality of the digitally produced guides is superior, which can be representative of the inherent benefits of the digital workflow. The 3D-printed and milled guides are both produced directly using the data of the virtual plan, which also avoids the steps of computer-controlled fabrication that cause an error in the conventional fabrication. The CBCT acquisition to virtual planning to guide fabrication has strict geometric fidelity and the error has restrictions to the resolution of the imaging modality, accuracy of the registration algorithms and dimensional tolerances of the fabrication technology [24]. Overall precision of the industrially milled guides was the best with a mean coronal and apical deviation of 0.38 mm and 0.52 mm, respectively. These values are lower than the accuracy range found in the literature on guided surgery and are close to the theoretical limits of sleeve-based guidance systems [25]. Five-axis CNC milling accuracy is well known in the dental prosthetics and manufacturing sector and tolerances of complex three-dimensional geometries can be achieved to tolerances of ± 0.02 to 0.05 mm [26]. Subtractive manufacturing process is dimensionally stable in inherent sense because the process of removing material of a pre-polymerized homogeneous blank does not entail phase-change distortions of additive manufacturing or thermoforming. The 3D-printed (desktop) guides provided intermediate precision with mean deviation values which were much better than conventional guides but a bit worse than milled guides. They found significant differences between printed and milled guides of apical deviation and angular deviation but not coronal-level measures or depth deviation which were statistically significant. This result indicates that the overall accuracy of 3D-printed guides is high, but the effect of magnification of errors, which are small angular corrections on the guide level, leading to an increasingly large deviation of the positions along the length of the implant, has a more significant impact at the apex of the implant than at the platform [27]. The lack of precision with desktop stereolithography printing has been described in various more recent studies and has been linked to a variety of factors. The layer-by-layer polymerization causes staircase artifacts which are more pronounced at rounded edges and at sharp features [28]. During curing of a polymer, contraction of the dimension up to 0.5 to 2.0 dimensions can be caused by polymerization shrinkage, which varies depending on the resin formulation and post-curing instructions [29]. The orientation of the build affects the accuracy greatly as sleeve orientation compared to the build platform affect the cylindricity and position accuracy of the guide channel. Nevertheless, the overall accuracy of the 3D-printed guides in the work was quite positive and in line with the recent reports that illustrated that modern desktop printers can produce guides accurate enough to be used in surgery [30]. The angular deviations of this study should be discussed in particular in regards to anterior maxillary implant placement. The direct clinical implication of the mean angular deviation of 4.87 with conventional guides versus 2.14 and 1.53 with 3D-printed and milled guides, in this case, respectively is obvious. Angular deviations of the anterior maxilla in turn translate to changed emergence of the implants and may also cause the screw-access channel to become located outside the cingulum region and may require angled abutments or cement-retained restorations, both of which reduce the optimal screw-retained prosthetic design [31]. The reduced angular deviations made possible by the use of digital guides help in ensuring more predictable screw-retained prosthetic results that are gaining popularity due to their maintenance benefits and lowered biological problems. Although being smaller in magnitude than the lateral deviations, the depth ones are clinically important since an excessive depth may put the implant platform below the optimum level of the prosthetic interface, whereas an insufficient depth may lead to a lack of primary stability or the revelation of rough surfaces to the implant [32]. The guided surgery sleeve system offers a mechanical depth stop that assists in controlling depth deviation in all types of guides and this is the reason why the magnitude of depth error is relatively lower than the magnitude of lateral error in all the groups. Nevertheless, the standard guides exhibited almost three times more depth deviation than milled guides, which indicates that the accuracy of sleeve positioning and guide stability in drilling influence depth control too. There are various limitations of this study that ought to be taken into account when explaining the results. *in vitro* design, in as much as it offers high standardization and control, fails to simulate the clinical complications of limited mouth opening, patient movement, saliva, bleeding, soft tissue interference and variable bone density in the real *in vivo* [33]. Singular operator usage could also be detrimental to generalizability, with operator experience and ability having an effect on surgical guide-dependent accuracy in clinical practice. The analysis of one location of implant in the central incisor does not reflect the possible differences in the accuracy of other anterior location or in partially versus fully edentulous arches. There was also a single 3D printer and a single milling system that were tested and the outcomes do not necessarily correspond to other systems that are provided commercially [34]. Future research ought to compare other fabrication technologies, investigate accuracy in clinical practice, determine whether guide support type has an effect on accuracy and determine the relationship between guide accuracy and fabrication investment cost [35].

## Conclusion:

Surgical guide fabrication method significantly influenced osteotomy accuracy in anterior maxillary implant sites, with digitally fabricated guides performing better than conventional vacuum-formed guides across all measured parameters. Industrially milled guides demonstrated the highest overall accuracy, while desktop 3D-printed guides also showed clinically acceptable precision and performed substantially better than conventional guides. Therefore, digitally fabricated surgical guides, particularly milled guides, are recommended for implant placement in the anterior esthetic zone where high positional accuracy is essential.

## References

[1] Nowicki AA, Markiewicz M (2025). J Clin Med..

[2] Heijtmeijer ST (2024). PeerJ..

[3] Kivovics M (2022). J Dent..

[4] Ballesteros J (2025). Clin Implant Dent Relat Res..

[5] Husain F (2024). J Indian Soc Periodontol..

[6] Mai HN (2023). J Med Internet Res..

[7] Yeager B (2024). J Prosthet Dent..

[8] Henprasert P (2020). J Prosthodont..

[9] Markiewicz M, Nowicki AA (2025). J Clin Med..

[10] Younis H (2024). Head Face Med..

[11] Spille J (2021). Int J Implant Dent..

[12] He SX (2024). BMC Oral Health..

[13] Yan K (2026). Aesthetic Plast Surg..

[14] Pessoa R (2022). J Prosthodont..

[15] Arunjaroensuk S (2024). J Dent Sci..

[16] Taheri Otaghsara SS (2023). J Dent..

[17] Bochet Q (2024). J Stomatol Oral Maxillofac Surg..

[18] Etajuri EA (2020). Dent Med Probl..

[19] Salem OAEH (2025). BMC Oral Health..

[20] Jin C (2025). J Dent..

[21] Yeung M (2020). J Prosthet Dent..

[22] Sun Y (2022). Clin Oral Implants Res..

[23] Chen X (2023). J Oral Maxillofac Surg..

[24] Al Kabany MH (2023). Int J Oral Maxillofac Implants..

[25] González-Rueda JR (2023). J Clin Exp Dent..

[26] Chen LW (2022). World J Clin Cases..

[27] Chen YJ (2026). J Prosthet Dent..

[28] Herstell H (2022). Int J Comput Dent..

[29] Wei SM (2021). Clin Oral Implants Res..

[30] Li H (2025). Beijing Da Xue Xue Bao Yi Xue Ban..

[31] Ochandiano S (2022). Front Oncol..

[32] Fan Y (2024). J Prosthodont..

[33] Mosch R (2025). Clin Exp Dent Res..

[34] Herschdorfer L (2021). J Prosthet Dent..

[35] D'haese R (2021). J Clin Med..

